# Allogeneic anti-CD19 CAR-T cells induce remission in refractory systemic lupus erythematosus

**DOI:** 10.1038/s41422-025-01128-1

**Published:** 2025-05-08

**Authors:** Chunmei Yang, Chuanyin Sun, Binghe Tan, Chao Hu, Liyan Wan, Chris Wang, Xiujuan Shi, Juliang Qin, Na Zhang, Biao Zheng, Mingyao Liu, Jin Lin, Bing Du, Hongyan Tong

**Affiliations:** 1 Department of Hematology, The First Affiliated Hospital, College of Medicine, Zhejiang University, Hangzhou, Zhejiang China; 2 Department of Rheumatology, The First Affiliated Hospital, College of Medicine, Zhejiang University, Hangzhou, Zhejiang China; 3 Shanghai Frontiers Science Center of Genome Editing and Cell Therapy, Shanghai Key Laboratory of Regulatory Biology and School of Life Sciences, East China Normal University, Shanghai, China; 4 BRL Medicine Inc., Shanghai, China; 5 Zhejiang Provincial Clinical Research Center for Hematologic Diseases, Hangzhou, Zhejiang China; 6 Zhejiang Province Key Laboratory of Hematology Oncology Diagnosis and Treatment, Hangzhou, Zhejiang China

**Keywords:** Autoimmunity, Cancer immunotherapy

Dear Editor,

Following the first clinical use of autologous CAR-T cell therapy in a patient with relapsed/refractory systemic lupus erythematosus (SLE), numerous clinical trials have been initiated to evaluate the safety and efficacy of CAR-T cell therapy in B cell-mediated autoimmune diseases.^1–5^ Allogeneic CAR-T cells, distinguished by their homogeneity, rapid availability, and potential for cost effectiveness, represent a promising therapeutic modality for autoimmune diseases. However, the risks of graft-versus-host disease (GVHD), allogeneic rejection, potential genotoxic stress associated with gene editing, and infection risk due to over-immunosuppression limit the broader clinical application of current allogeneic CAR-T cell products.^6^ In this study, we demonstrated that an allogeneic CD19-targeted CAR-T cell therapy is effective and safe in treating relapsed/refractory SLE, irrespective of prior neuropsychiatric involvement, using a reduced-intensity lymphodepletion regimen.

This investigator-initiated, single-center pilot study (NCT05988216) assessed the safety and efficacy of allogeneic anti-CD19 CAR-T cells (TyU19) in refractory SLE, following approval by the Medical Ethics Committee of The First Affiliated Hospital, Zhejiang University. The generation of TyU19 has been previously described.^7^ All enrolled SLE patients met pre-defined inclusion and exclusion criteria (Supplementary information, Methods). Between Sep 2023 and Sep 2024, four young women (aged 22–24 years) with refractory SLE were enrolled, presenting baseline SELENA-SLEDAI scores ranging from 14 to 26. All patients had a history of multi-organ involvement, including lupus cerebritis (S01 in 2018, S02 in 2019, S03 in 2021), but presented without active central nervous system lupus at enrollment. Prior therapies included various immunosuppressants and biologics (Supplementary information, Table S1).

The treatment scheme is depicted in Fig. 1a. Patients S01, S02, and S03 received lymphodepletion chemotherapy consisting of intravenous fludarabine (25 mg/m^2^ body surface area per day) on days 5, 4, and 3, and cyclophosphamide (300 mg/m^2^ body surface area per day) on days 5 and 4. All patients received an infusion of 1 × 10^6^ CAR^+^ T cells/kg of patient body weight on day 0. Prior to enrollment, patient S04 was treated with telitacicept, necessitating a 6-week washout period. However, disease progression occurred during this washout, manifesting as worsening arthralgia, chest tightness, and low-grade fever, requiring treatment with cyclophosphamide (600 mg) to control disease. Following an additional two-week washout period, without further lymphodepletion, CAR-T cells were directly infused.

**Fig. 1:**
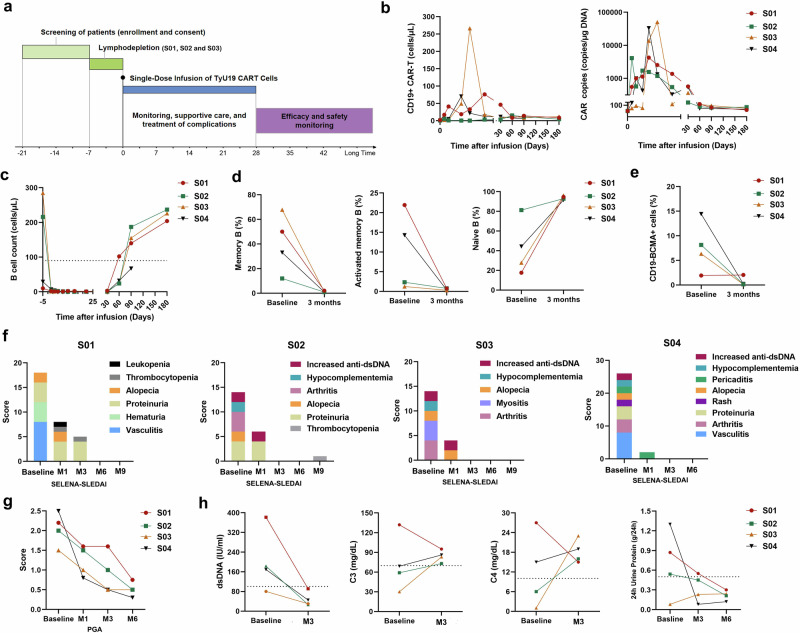
Expansion of TyU19, reconstitution of B cells and efficacy of TyU19 intervention for SLE. **a** Therapeutic timeline for treating patients with SLE by TyU19 cells. **b** Circulating CAR^+^ cells were detected by flow cytometry. The CAR copy numbers were determined by quantitative PCR. **c** Numbers of circulating B cells were analyzed within 6 months after infusion. **d**, **e** Changes in percentages of memory B cells (CD21^+^CD27^+^), activated memory B cells (CD11c^+^CD21^lo^), naïve B cells (CD21^+^CD27^−^) and CD19^−^BCMA^+^ plasma cells in peripheral blood mononuclear cells at 3 months after infusion. **f**, **g** SELENA-SLEDAI and PGA scores were assessed in four patients after TyU19 cells administration. **h** Changes of anti-dsDNA antibodies, complement factor C3, complement factor C4, and 24h urine protein after TyU19 cells infusion.

TyU19 demonstrated robust in vivo expansion, peaking between days 7 and 14, and dropped to a very low level at 2 months after the transfusion (Fig. 1b). Infusion induced rapid B-cell depletion within one week, followed by reconstitution of a predominantly naïve B-cell population with a paucity of memory B cells (Fig. 1c, d). A significant enrichment of CD19^–^BCMA^+^ cells was observed in patients S02, S03 and S04. However, following TyU19 infusion, the CD19^–^BCMA^+^ cells and CD19^–^CD138^+^ cells showed a significant decline by 3 months (Fig. 1e; Supplementary information, Fig. S1a).

All four patients demonstrated sustained improvement in clinical signs and symptoms. At 3-month follow-up, all patients met the Systemic Lupus International Collaborating Clinics (SLICC) criteria for a sustained response (SRI-4). All patients achieved a SELENA-SLEDAI score of zero and a PGA score of <1 at 3–6 months (Fig. 1f, g). Symptoms of arthritis (patients S02, S03 and S04), alopecia (patients S01, S02, S03 and S04), and finger vasculitis/ulceration (patients S01 and S04) all resolved. Complement factors C3 and C4 became normal within one month in all patients. Anti-dsDNA antibody levels decreased and proteinuria also resolved in all patients (Fig. 1h). The levels of antibodies against Smith (Sm) antigen, U1 small nuclear ribonucleoprotein (U1-RNP), and Ro52 showed a marked decrease (Supplementary information, Fig. S1b–d). Importantly, patient S01 discontinued all immunomodulatory and immunosuppressive medications, including glucocorticoids, at 3 months, achieving drug-free remission. The other three patients currently are still undergoing maintenance therapy with low-dose corticosteroids. Employing a low-dose corticosteroid regimen with gradual tapering has the potential to sustain and consolidate previously attained clinical benefits. The most commonly observed grade 3 or 4 adverse events within the first few weeks were neutropenia, lymphopenia, hepatic dysfunction, fever, and fatigue (Supplementary information, Tables S2, S3). Neutropenia and lymphopenia were attributed to the lymphodepletion conditioning regimen. All patients experienced only grade 1 cytokine release syndrome (CRS), manifesting as fever that persisted for 2–3 days (Supplementary information, Fig. S2a, b). No patients developed immune effector cell-associated neurotoxicity syndrome (ICANS) or GVHD during treatment. Cytokine (Supplementary information, Fig. S3) and C-reactive protein (CRP) levels (Supplementary information, Fig. S2c) were closely monitored and did not exhibit significant elevations. Patients S02, S03 and S04 experienced a decrease in IgA, IgG, and IgM levels after CAR-T cell infusion (Supplementary information, Fig. S2d–f). A comparative analysis of hepatitis B antibody titers, performed before CAR-T cell infusion and at 3 months post-infusion, demonstrated no substantial diminution in vaccination responses, aligning with findings reported by Mackensen et al.^2^ (Supplementary information, Fig. S2g). This maintaining of hepatitis B antibody after CD19 CAR-T cell infusion seems very interesting, which needs to be further investigated in the future study. All patients remained infection-free during their hospitalization and follow-up periods. Acyclovir and sulfamethoxazole were administered orally until B-cell recovery.

This study reports the first clinical application of allogeneic anti-CD19 CAR-T therapy in patients with refractory SLE, demonstrating significant clinical remission and a favorable safety profile, thus expanding the potential of allogeneic CAR-T therapy in treating autoimmune diseases. To mitigate the risk of host immune rejection of allogeneic CAR-T cells, a more rigorous lymphodepletion regimen is typically employed prior to infusion compared to autologous CAR-T therapy. This often involves the administration of anti-CD52 antibodies, resulting in enhanced immunosuppression and a heightened incidence of adverse events, particularly severe infections.^8–10^ However, the allogeneic CAR-T cells (TyU19) used in this trial were systematically modified by CRISPR/Cas9 to disrupt the expression of TRAC, HLA-A, HLA-B, CIITA as well as PD-1. These modifications were previously shown to have excellent safety and efficacy in treating relapsed/refractory severe myositis and systemic sclerosis.^7^ This allowed for the implementation of a reduced-intensity lymphodepletion regimen compared to that typically used for CAR-T therapy in hematologic malignancies and autoimmune disorders. Despite this reduction, TyU19 cells exhibited dramatic expansion and persistence for over two months accompanied by grade 1 CRS, implying that the modification of allogeneic CAR-T cells is sufficient to avoid immune rejection.

The hallmark of SLE is the presence of autoantibodies generated by aberrant plasma cells and B cells.^11,12^ Long-lived plasma cells (LLPCs), enriched within the CD19^–^CD38^hi^CD138^hi^ plasma cell subset, are an important source of autoantibody production in SLE.^13^ The lack of CD19 in LLPCs raises questions about the applicability of CD19 CAR-T therapy in autoantibody-mediated autoimmune diseases.^14^ Interestingly, although a significant portion of CD19^–^BCMA^+^ cells were observed in patients S02, S03 and S04 prior to TyU19 infusion, all patients experienced remarkable clinical improvements following therapy. Notably, all patients exhibited a decline in peripheral BCMA^+^ as well as CD19^–^BCMA^+^ plasma cells, suggesting that TyU19 infusion may facilitate the reconstitution of plasma cells.

Consistent with observations in autologous CAR-T cell therapy, the majority of reconstituted B cells were naïve B cells, with a drastic reduction in memory B cells and plasma cells. Of note, one patient in this cohort who discontinued pharmacologic intervention remains in sustained, medication-free remission, highlighting the potential advantages of allogeneic CAR-T cells as a promising therapeutic approach for patients with refractory SLE. Further research is warranted to explore its long-term efficacy and optimize its application in this challenging disease.

## Supplementary information

Supplementary file
