# Supplementary material for: Allogeneic anti-CD19 CAR-T cells induce remission in refractory systemic lupus erythematosus
Source: Cell Res. 2025 May 8;35(8):607–9. doi: 10.1038/s41422-025-01128-1 (PMC12297247; doi:10.1038/s41422-025-01128-1)
Supplement: Supplementary file 1 — Supplementary file [file 41422_2025_1128_MOESM1_ESM.pdf]

Supplementary information, Methods

Generation of allogeneic anti-CD19 CAR-T cells

In brief, the second-generation 4-1BB contained CD19 CAR together with trancked extracellular domain of PD-L1 (PD-L1 ECD) was delivered into health donor derived T cells by third-generation lentivirus. Then the TRAC, HLA-A, HLA-B, CIITA as well as PD-1 was modified by transient Cas9/gRNA (RNP) complex electroporation through electroporation (MaxCyte). Finally, the CAR positive rate and gene knockout efficiency was analyzed by flow cytometry for quality control.

Inclusion and exclusion criteria of SLE patients

Inclusion criteria:

1. Age of 18–65 years (inclusive), male or female;

2.Patients diagnosed with SLE according to the 2019 European League Against Rheumatism/American College of Rheumatology (EULAR/ACR) classification criteria for SLE;

ANA ≥ 1:80, or test positive for anti-dsDNA antibody and/or anti-Sm antibody;

3. No response to conventional treatment or recurrence of disease activity after disease remission. Definition of conventional treatment: use of two or more drugs including glucocorticoids (> 1 mg/kg/day) and any one or more of the following immunomodulatory drugs for more than 6 months: antimalarials, cyclophosphamide, azathioprine, mycophenolate mofetil, methotrexate, leflunomide, tacrolimus, cyclosporine, and biologics including rituximab, belimumab, and telitacicept.

4. At least one category A or two category B organ assessments according to the

British Isles Lupus Assessment Group 2004 index (BILAG-2004), or both;

5. SELENA-SLEDAI score ≥ 8;

6. Positive expression and expression rate of CD19 on peripheral blood B cells determined by flow cytometry;

7. The functions of vital organs meet the following requirements:

1) Bone marrow hematopoietic function:

a. White blood cell count ≥ 3 × 109/L;

b. Neutrophil count ≥ 1 × 109/L (no colony-stimulating factor treatment within 2 weeks prior to examination);

c. Platelets ≥ 50 × 109/L;

d. Hemoglobin ≥ 80 g/L

2) Liver function: alanine aminotransferase (ALT) ≤ 3 × ULN; aspartate aminotransferase (AST) ≤ 3 × ULN; total bilirubin (TBIL) ≤ 1.5 × ULN (except for Gilbert syndrome, total bilirubin ≤ 3.0 × ULN);

3) Renal function: creatinine clearance (CrCl) ≥ 60 mL/min (Cockcroft/Gault formula)

4) Coagulation function: international normalized ratio (INR) ≤ 1.5 × ULN, prothrombin time (PT) ≤ 1.5 × ULN.

5) Cardiac function: good hemodynamic stability and left ventricular ejection fraction (LVEF) ≥ 55%;

8. Female subjects of childbearing potential and male subjects whose female partners are of childbearing potential should adopt medically recognized contraceptive measures or keep abstinence during the study treatment and within at least 6 months

after the end of study treatment; female subjects of childbearing potential should have a negative serum HCG test

result within 7 days before study enrollment and be not in lactation;

9. Voluntarily participate in this clinical study, sign the informed consent form, have good compliance, and cooperate with follow-up visits.

Exclusion criteria:

1. Subjects with a history of severe drug allergy or allergic constitution;

2. Presence of or suspected presence of fungal, bacterial, viral infections or other infections that cannot be controlled or require intravenous therapy;

3. Central nervous system disorders caused by SLE or disorders other than SLE (including epilepsy, psychiatric disorders, organic encephalopathy syndrome, cerebrovascular accident, encephalitis, and central nervous system vasculitis);

4. Patients with severe cardiac disorders (e.g., angina pectoris, myocardial infarction, heart failure, and arrhythmia);

5. Subjects with congenital immunoglobulin deficiency;

6. Subjects with other malignancies (except non-melanoma skin cancer and *in situ* cervical cancer, bladder cancer, and breast cancer in subjects whose disease-free survival is more than 5 years);

7. Subjects with end-stage renal failure;

8. Have received any of the following SLE treatments:

1) Have received therapeutic doses of corticosteroids (defined as > 20 mg/day of prednisone or equivalent) prior to enrollment or within 72 h before BRL-301 infusion.

2) Have received any other clinical study drugs for SLE within 4 weeks prior to enrollment. However, enrollment is permitted if the study treatment was ineffective or disease progression occurred during the study treatment period and at least 3 half-lives have elapsed before enrollment.

3) Have received anti-CD20 monoclonal antibody (such as rituximab) within 4 weeks, telitacicept within 6 weeks, and belimumab within 12 weeks prior to screening.

4) Previously received CAR-T cell or other genetically modified T cell therapies.

9. Subjects with hepatitis B surface antigen (HBsAg) or hepatitis B core antibody (HBcAb) positive and peripheral blood HBV DNA titer higher than the upper limit of detection; subjects with hepatitis C virus (HCV) antibody positive and peripheral blood HCV RNA positive; subjects with human immunodeficiency virus (HIV) antibody positive; syphilis test positive;

10. Subjects with psychiatric disorders and severe cognitive dysfunction;

11. Subjects who participated in other clinical trials within 3 months before enrollment;

12. Females who are pregnant or planning to become pregnant;

13. Subjects who are unsuitable for being included into this study as deemed by the investigator due to other reasons.

The donor selection and inclusion criteria.

The donor selection and inclusion criteria received approval from the Medical Ethics Committee of The First Affiliated Hospital, Zhejiang University (IIT20210001C-D2). All participants were volunteers and provided their informed consent. A detailed

exposition of the inclusion criteria is provided below:

1. Voluntary participation in this blood donation program, with a signed informed consent form.

2. Volunteers aged between 18 and 35 years old.

3. Body weight requirements: males ⩾ 50kg, females ⩾ 45kg.

4. Blood pressure norms:

a) Systolic blood pressure between 12.0kPa (90mmHg) and 18.7kPa (140mmHg);

b) Diastolic blood pressure between 8.0kPa (60mmHg) and 12.0kPa (90mmHg);

c) Pulse pressure difference of at least 30mmHg or 4.0kPa.

5. Pulse rate of 60–100 beats/min, or ⩾ 50 beats/min in highly trained endurance athletes, with a regular rhythm.

6. Normal body temperature.

7. General physical health that satisfies the following:

a) Healthy cardiovascular and pulmonary function, with no abnormal breath sounds or heart murmurs. Heart rate is between 60–100 beats/min, and chest X-rays show no significant abnormalities;

b) Abdomen should be soft with no tenderness, masses, or organ enlargement of the liver and spleen;

c) Clear skin with no infections, jaundice, or extensive dermatological conditions; no significant enlargement of superficial lymph nodes;

d) No severe illnesses of sensory organs, no jaundiced sclera, and no thyroid gland enlargement;

e) Limbs must be free from major disabilities, severe functional issues, or swollen/red joints;

f) Both arms' venipuncture sites should be intact, without skin lesions or signs of intravenous drug use.

8. Normal hemoglobin levels: ⩾ 120g/L for males, ⩾ 115g/L for females (using the copper sulfate method: male ⩾ 1.0520, female ⩾ 1.0510).

9. Biochemical laboratory tests indicate normal liver and kidney functions:

a) Liver enzymes: ALT and AST;

b) Renal tests: creatinine, urea, uric acid.

10. No serological evidence of infections for viruses such as: hepatitis B (HBV), hepatitis C (HCV), HIV, Epstein-Barr virus (EBV), cytomegalovirus (CMV), and syphilis.

11. No history of receiving allogeneic cellular therapies, whole blood transfusions, blood components transfusions, bone marrow or organ transplants.

12. No prior severe medical conditions.

13. No history of noticeable reactions or complications from previous blood donations.

14. A minimum six-month interval since the last blood donation prior to participating in this study.

15. Full compliance with the rules outlined for this blood donation project.

Clinical response assessment

Each patient underwent a systematic follow-up assessment of efficacy and safety after

the infusion of TyU19. Following CAR-T cell infusion, SLE disease activity was assessed by means of the score on the Safety of Estrogens in Lupus Erythematosus National Assessment-Systemic Lupus Erythematosus Disease Activity Index (SELENA-SLEDAI), British Isles Lupus Assessment Group 2004 (BILAG-2004) index, Physician Global Assessment (PGA), and Systemic Lupus Erythematosus Responder Index-4 (SRI-4). In addition, levels of urinary protein excretion, complement factor C3, complement factor C4 and double-stranded DNA (dsDNA) antibodies were assessed.

Toxicity evaluation

After CAR-T cell infusion, patients will be closely monitored for the occurrence of cytokine release syndrome (CRS), immune effector cell-associated neurotoxicity syndrome (ICANS), and graft-versus-host disease (GVHD). CRS and ICANS will be evaluated according to the consensus criteria of the American Society for Transplantation and Cellular Therapy. Any signs of acute GVHD will be monitored based on the 2016 Mount Sinai criteria, and signs of chronic GVHD will be monitored following the 2020 NCCN guidelines. Other AEs were evaluated according to the Common Terminology Criteria for AE (CTCAE v. 4.03).

Flow cytometry

CAR and cell surface marker expression were determined using flow cytometry. Cells were pre-washed and incubated with antibodies for 30 min on ice. After two washes, the samples were run on an LSR Fortessa (BD Biosciences, NJ, USA) and analyzed using Flow Jo software. The following antibodies were used, anti-human CD3, CD4,

CD8, CD19, BCMA, HLA-A, HLA-B, HLA-DR and carboxyfluorescein diacetate succinimidyl ester (CFSE) (BD Biosciences). To detect CAR expression, FITC-conjugated human CD19 (20-291) protein (ACRO biosystems, CD9-HF2H2 and CD9-HF251) were added sequentially.

qPCR analysis of CAR-T cells expansion

Blood samples were collected before and after CAR-T cell infusion. Lysis Buffer (BD Biosciences) was first added and genomic DNA was acquired using Genomic DNA Purification Kit (ThermoFisher). TaqMan qPCR assay was performed to measure CAR copy number in peripheral blood cells. qPCR was run on a QuantStudio™ 3 Real-Time PCR System (ThermoFisher). Each sample was determined in triplicate.

Supplementary information, Table S1 Patient characteristics at baseline

	S01	S02	S03	S04
Age (years)	33	25	22	24
Gender	Female	Female	Female	Female
Disease duration (years)	11	7	8	6
Disease activity SELENA-SLEDAI (score)	18	14	14	26
**Laboratory values**				
Baseline C3 (mg/dL)	132	59	30	69
Baseline C4 (mg/dL)	27	6	1	15
Baseline anti-dsDNA (IU/mL)	80.31	381.73	183.07	169.13
Baseline ANA	1:320	1:160	1:320	1:320
Proteinuria (g/24h)	0.87	0.54	0.08	1.3
**Organ involvement (* Representing organ involvement before CAR-T cell infusion)**				
Skin	+*	+*	+*	+*
Joint	+*	+*	+*	+*
Kidney	+*	+*	+	+*
Nervous system	+	+	+	
Gastrointestinal			+	
Cardiorespiratory system				+*
Hematological	+	+*	+	
**Treatments (# Representing the drugs used before CAR-T cell therapy)**				
Glucocorticoids	+#	+#	+#	+#
Hydroxychloroquine	+#		+#	
Mycophenolate mofetil			+#	+#
Cyclophosphamide	+	+		+
Tacrolimus	+#	+#	+	
Belimumab	+		+	+
Telitacicept		+		+#
Other	Cyclosporine	Azathioprine	Rituximab	

C3, complement factor c3; C4, complement factor c4; dsDNA, double-stranded DNA； ANA, antinuclear antibody; UPCR, urine protein creatinine ratio.

Supplementary information, Table S2 Adverse Events in all three patients who received TyU19

Adverse events N (%)	Grade1	Grade 2	Grade 3	Grade 4
CRS	4 (100%)	0 (0%)	0 (0%)	0 (0%)
Lymphocytosis	0 (0%)	0 (0%)	2 (50%)	2 (50%)
Granulocytopenia	1 (25%)	0 (0%)	2 (50%)	1 (25%)
Anaemia	0 (0%)	1 (25%)	3 (75%)	0 (0%)
Thrombocytopenia	0 (0%)	1 (25%)	0 (0%)	0 (0%)
Hypoalbuminaemia	1 (25%)	1 (25%)	0 (0%)	0 (0%)
Hypocalcaemia	0 (0%)	1 (25%)	0 (0%)	0 (0%)
Hypertriglyceridaemia	2 (50%)	0 (0%)	0 (0%)	0 (0%)
Hypokalaemia	1 (25%)	0 (0%)	0 (0%)	0 (0%)
Elevated LDH	2 (50%)	0 (0%)	0 (0%)	0 (0%)
Elevated ferritin	1 (25%)	0 (0%)	0 (0%)	0 (0%)
Elevated CRP	1 (25%)	2 (50%)	0 (0%)	0 (0%)
Elevated ALT	0 (0%)	0 (0%)	1 (25%)	0 (0%)
Elevated AST	0 (0%)	0 (0%)	1 (25%)	0 (0%)
Elevated γ-GT	0 (0%)	1 (25%)	0 (0%)	0 (0%)
Elevated ALP	1 (25%)	0 (0%)	0 (0%)	0 (0%)
pyrexia	1 (25%)	3 (75%)	0 (0%)	0 (0%)
Edema lower limb	0 (0%)	1 (25%)	0 (0%)	0 (0%)
Rash	1 (25%)	0 (0%)	0 (0%)	0 (0%)
cough	1 (25%)	0 (0%)	0 (0%)	0 (0%)
Myalgia	1 (25%)	0 (0%)	0 (0%)	0 (0%)
Pain knee	0 (0%)	1 (25%)	0 (0%)	0 (0%)

CRS cytokine release syndrome; LDH lactate dehydrogenase; CRP C-reactive protein; ALT alanine aminotransferase; AST aspartate aminotransferase; γ-GT γ-glutamyl transferase; ALP alkaline phosphatase

Supplementary information, Table S3 Safety of TyU19 cell therapy in SLE

	S01	S02	S03	S04
CRS Grade	1	1	1	1
ICANS Grade	0	0	0	0
Bone marrow toxicity [3]	0	0	0	0
Tocilizumab treatment	0	0	0	+
Glucocorticoid treatment	0	+	0	+
Low IgG	0	+	+	+
IgG substitution	0	0	0	0

CRS Cytokine Release Syndrome; ICANS Immune Effector Cell-associated Neurotoxicity Syndrome.

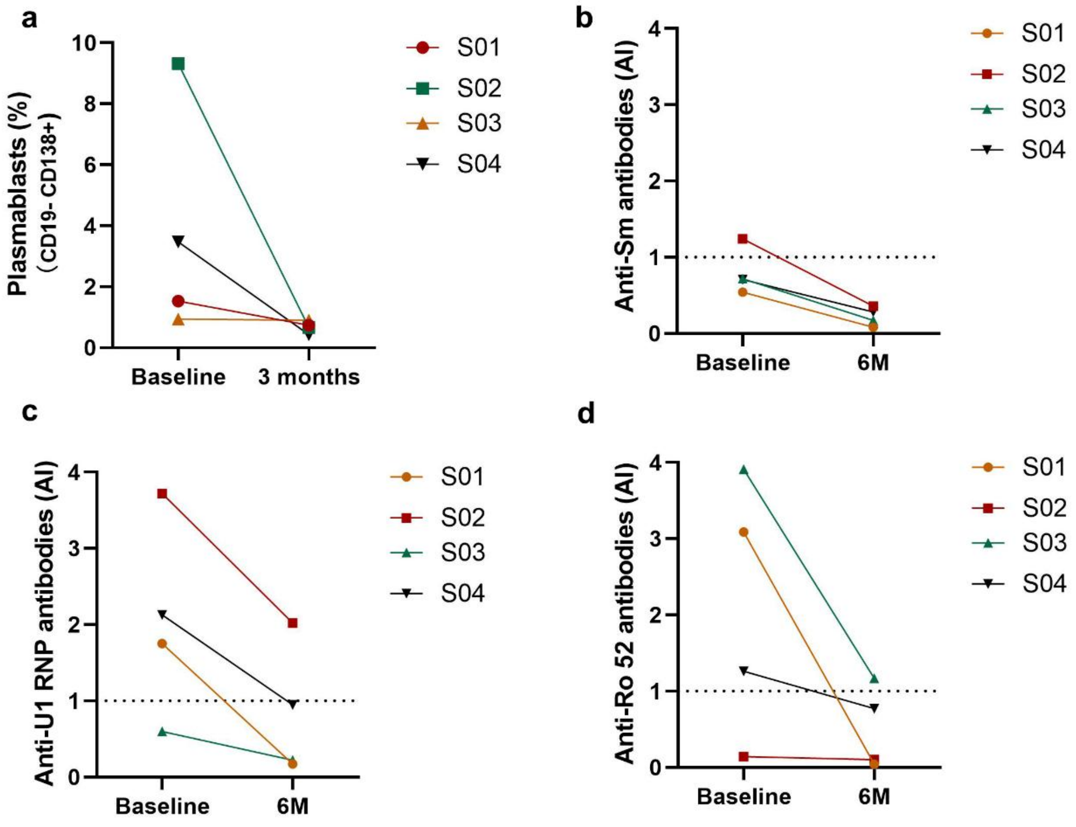

Supplementary information, Fig. S1 a. Changes in the numbers of CD19-CD138+ plasma cells in PBMC at 3 months after infusion; b-d Changes of anti-Sm antibody (b), anti-U1-RNP (c) and anti-So 52 (d) after TyU19 cells infusion.

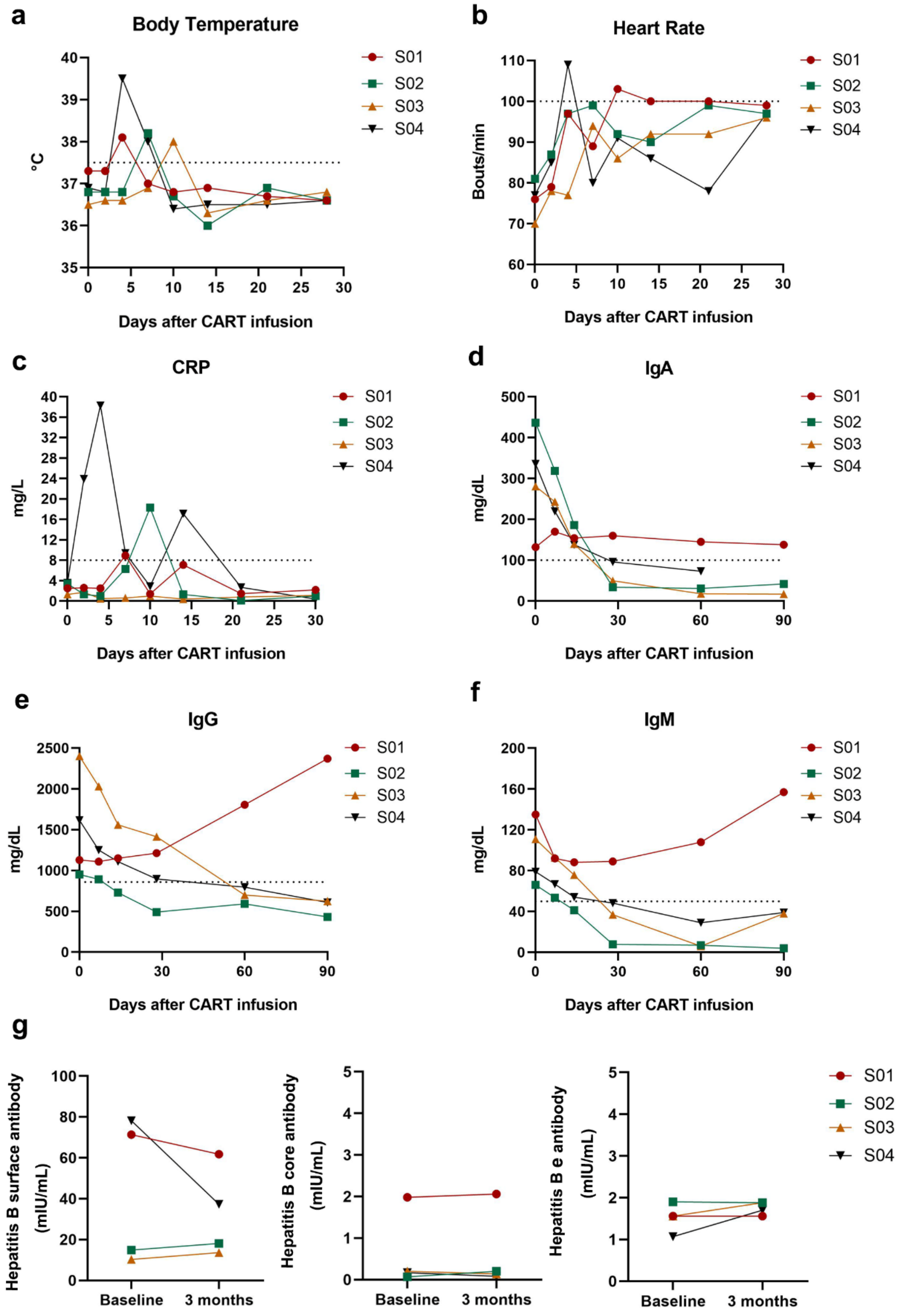

Supplementary information, Fig. S2 Safety of TyU19 treatment in SLE. Body temperature (a), heart rate (b), serum levels of c-reactive protein (c) during the first

30 d after CAR T cell administration; d-f. Trends in serum immunoglobulins including IgG, IgA, and IgM after CAR-T cell infusion; g. Antibody levels against hepatitis B surface antigen, hepatitis B core antigen and hepatitis B e antigen were assessed at baseline and three months after CAR T cell therapy administration.

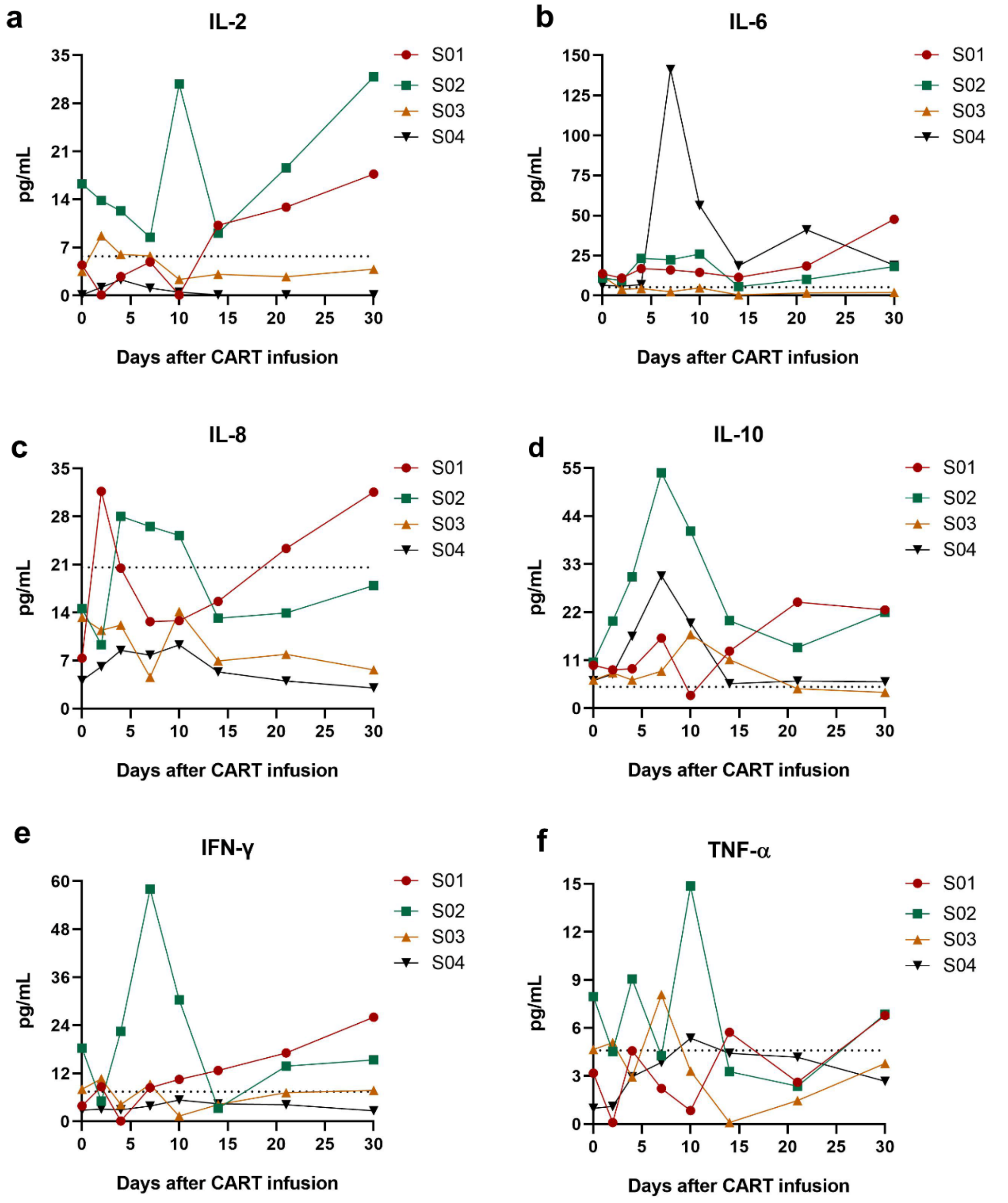

Supplementary information, Fig. S3 Changes in serum levels of cytokines, interleukin (IL)-2, IL-6, IL-8, IL-10, TNF-α and IFN-γ.
